# New Axially Expandable Oblique Cage Designed for Anterior to Psoas (ATP) Approach: Indications-Surgical Technique and Clinical-Radiological Outcomes in Patients with Symptomatic Degenerative Disc Disease

**DOI:** 10.3390/jcm13123444

**Published:** 2024-06-12

**Authors:** Massimo Miscusi, Sokol Trungu, Luca Ricciardi, Stefano Forcato, Antonella Mangraviti, Antonino Raco

**Affiliations:** 1 Department of Neurosurgery, Sant’Anna University Hospital, 44121 Ferrara, Italy; 2 Neurosurgery Unit, Cardinale G. Panico Hospital, 73039 Tricase, Italy; 3 NESMOS Department, Sant’Andrea Hospital, Sapienza University of Rome, 00185 Rome, Italy

**Keywords:** OLIF, expandable cage, anterior lumbar approaches, lumbar degenerative disk disease

## Abstract

**Background**: Standard oblique cages cannot cover endplates side-to-side, which is an important biomechanical factor for reducing the risk of cage subsidence and for restoring correct segmental lordosis. The aim of this study is to evaluate the radiological and clinical results of a new oblique lumbar interbody fusion (OLIF) axially expandable cage. **Methods**: This is a prospective observational case–control study. From March 2018 to June 2020, 28 consecutive patients with lumbar degenerative disease underwent an ATP approach, with the insertion of a new axially expandable cage, which was used as a stand-alone procedure or followed by posterior percutaneous pedicle fixation. **Results**: Twenty-eight patients in both groups met the inclusion criteria. The mean follow-up time was 31.2 months (range of 13–37). The clinical results were not significantly different, although in the control group, two major intraoperative complications were recorded, and slight improvements in ODI and SF-36 scores were observed in the study group. The radiological results showed a less frequent incidence of subsidence and a higher rate of fusion in the study group compared to controls. **Conclusions**: The axially expandable oblique cage for lumbar inter body fusion, specifically designed for the ATP approach, represents an innovation and a technical improvement. The insertion and the axial expansion technique are safe and easy. The large footprint could obtain solid and effective arthrodesis, potentially reducing the risk of subsidence.

## 1. Introduction

Different lumbar fusion procedures have been progressively accepted as the standard surgical treatment for many different spine conditions, such as degenerative disc disease (DDD), deformities, traumatic injuries, and spinal instability [1,2,3,4,5]. Lumbar discopathy is the most common, and different causes lead to degeneration within the intervertebral disc. In recent years, wearable inertial sensors have been used in the measurement of human gait analysis to evaluate pathological pathways [6,7].

Although standard posterior approaches have been systematically preferred in the past, the advent of minimally invasive surgery (MIS) has allowed to both ameliorate the surgical–clinical outcomes of posterior approaches and consider more feasible lateral, oblique, and anterior approaches to the thoraco-lumbar spine in properly selected cases [8,9,10]. While MIS in posterior surgeries has drastically reduced the rate of surgical injury to spine muscles and tendons, intraoperative blood loss, and the infection rate, the lateral, oblique, and anterior approaches have benefitted the most from the advent of MIS-dedicated retraction systems [5]. In fact, retraction systems, ranging from tubular devices to self-retaining multiple blades, allow the use of small skin incisions and the use of anatomical corridors to the spine column in anterior–oblique approaches, while a trans-psoas corridor is used for lateral procedures [11,12,13]. However, a review of larger studies in the literature is contradictory and showed no significant differences in larger population groups. A recent editorial by Chapman et al. concluded that MIS remains an interesting care option but is far from being a new “standard of care” [14].

The anterior-to-psoas (ATP) approach is a MIS procedure consisting of an oblique approach to the lumbar spine, which does not require the use of intraoperative monitoring since it is anterior-to-psoas by definition [15]. Nevertheless, the insertion of a cage through the aorto-psoas window may be technically demanding in terms of surgical dissection, vessel management, and implant positioning [15,16]. There are oblique cages specifically designed to enter the aorto-psoas corridor with the minimum retraction of the psoas and great vessels on the market [17]. Conversely, their shape and dimension are not able to entirely cover the epiphysial ring of the vertebral body, thus reducing the footprint area. Accordingly, an in-situ expansion of the implant is expected to overcome this limitation, eventually exploiting the aforementioned advantages while increasing the footprint area, the segmental deformity correction grade, and the chances for fusion and reducing the risk for subsidence [3].

The aim of this study was to evaluate the clinical–radiological outcomes in a single-center case series of patients affected by primary DDD, treated with an ATP approach using a new oblique, axially expandable cage.

## 2. Materials and Methods

### 2.1. Study Design 

This is a prospective observational cohort study from a single tertiary academic center. According to the study design and the non-modification of the standard of care, IRB approval was not required. All of the patients expressed written consent to undergo the surgical procedure after receiving appropriate information. The data reported have been completely anonymized. Therefore, this study is perfectly consistent, in all of its aspects, with the WMA Helsinki Declaration of Human Rights.

### 2.2. Patients’ Population 

Study group: From March 2018 to June 2020, 28 consecutive patients with degenerative lumbar disease who underwent pure anterior interbody lumbar fusion, using an ATP approach, with or without the supplementation of posterolateral instrumentation, were considered for eligibility. 

The inclusion criteria were as follows: the diagnosis of symptomatic mono or plurisegmental primary lumbar degenerative disc disease (DDD); unresponsiveness to conservative therapy for over 6 months before surgery; clinical–radiological follow-up (FU) longer than 6 months.

The exclusion criteria were as follows: spondylolisthesis greater then grade I according to Meyerding et al. [18]; severe stenosis grade C and D (defined by a neuroradiologist based on a classification on an MRI as described by Schizas et al.) [19]; unfavorable anterior vascular anatomy; unbalanced thoraco-lumbar deformities; active infection or malignancy; spine trauma or retroperitoneal surgery prior to the current hospitalization. 

Control group: A series of 28 matched patients, treated previously by the same surgeons at the same institution, for the same inclusion criteria, using a standard, non-expandable oblique cage (Avila, Medtronic, Minneapolis, USA), was considered as the control group in the present study. 

### 2.3. Surgical Technique 

The patient is positioned in the right lateral decubitus position. The skin incision is five cm (from L1-L2 to L4-L5) and 2 cm (for L5-S1) anterior to the ventral profile of the target disc. Abdominal wall muscles are dissected using blunt scissors, cotton pads, and fingers; then, the retroperitoneal space is progressively exposed by anteriorly mobilizing the peritoneal content. The psoas muscle is identified, and the first blunt retractor is carefully positioned anterior to its tendon, constituting the posterior limit of the surgical corridor. The ureter is generally mobilized anteriorly, together with the peritoneum content. Once the fat tissue of prevertebral plane is dissected, another two or three blunt retractors are positioned to expose the disk space and then fixed with pins to the upper and lower vertebral bodies. From L1 to L4, the disk is exposed through the aorto-psoas space, while the L5-S1 disc is usually exposed medially lower to the great vessel carrefour, then between the vessels’ bifurcation. Segmental vessels are usually not ligated unless they limit the surgical exposure or their traction grade is higher than recommended. The discectomy is carefully conducted, properly achieving endplates’ preparation without injuring the cortical rim though. A phantom is firstly used for the implant sizing; then, the selected 3D-printed porous titanium expandable cage (Tsunami Medical srl, Modena, Italy) is fully filled with bone matrix (Attrax—Nuvasive, San Diego, CA, USA) and positioned in the discal space under fluoroscopic guidance using the dedicated driver. In the L-L view, the cage is positioned in the medial third of the disc space; in the A-P projection, the cage is pushed contralaterally until its lateral border reaches the contralateral epiphyseal ring limit, and then, the cage is reversely expanded, up to reaching the ipsilateral epiphyseal ring. A drainage is left in the retroperitoneal space. No lumbar orthosis is used after surgery. 

### 2.4. Clinical and Radiological Outcomes

General and neurological conditions, as well as the quality of life, were evaluated at the hospitalization time (t-0) as baseline data, and at 1-year follow-up (FU) (t1), using patients’ reported outcomes measurements (PROMs), such as the ten-point itemized visual analog scale (VAS) for back pain, the Oswestry Disability Index (ODI), and the short-form SF-36 score. The following radiological exams and measurements were collected at t-0 and t-1: lumbar standing and dynamic X-rays, to evaluate the segmental alignment and instability, respectively. The following parameters were considered for the spinal alignment evaluation: lumbar lordosis (LL), segmental lordosis (SL), segmental coronal cobb angle (SCCA), and PI-LL mismatch. MRI and CT were used to investigate the course of retroperitoneal great vessels, the foraminal height (FH), and the disk height (DH) at the treated level. The final length of the cage after its expansion on the latero-lateral plane, the presence of implant subsidence (loss of interbody space height during follow-up), and cage dislocation were also evaluated on the postoperative CT scan and standing X-rays. Segmental fusion was evaluated on a 12-month postoperative CT scan using the criteria described by Proietti et al. [20]. Intra- and perioperative complications have been recorded.

### 2.5. Statistical Analysis

Values were reported as mean ± standard deviation. The *t*-Student test was used to compare the quantitative continuous variables. Fisher’s exact test (2-sided) was used instead to compare the categorical variables. Statistical significance was pre-determined at an alpha value of 0.05. AnalystSoft Inc., StatPlus 2020 © (AnalystSoft Inc., Brandon, FL, USA) was used for data analysis.

## 3. Results

### 3.1. Patients and Operative Results

A total of 28 patients underwent an ATP approach with an expandable cage during the study period and met the inclusion criteria for our study. There were 12 (42.9%) women and 16 (57.1%) men. The mean age at the time of surgery was 64.2 ± 7.2 years (range 42–81). The presenting symptoms were lower back pain (100%) and radiculopathy (39.3%). The most common co-morbidity was cardiovascular diseases (60.7%), followed by diabetes mellitus (35.7%), obesity (28.6%), and respiratory diseases (21.4%). Thirteen patients (46.4%) were smokers. The most common level of interest was L4-L5 (41.7%), followed by L3-L4 (33.3%), L5-S1 (16.7%), and L2-L3 (8.3%). Nineteen patients (67.8%) underwent single-level, six patients (21.4%) underwent two-level, and three patients (10.7%) three-level OLIF, respectively. The mean length of surgery was 89 ± 14.8 min (range 60–210) with an average of 100 ± 16.2 mL (range 40–200 mL) of estimated blood loss (EBL). The mean length of stay (LOS) was 2 days (range 1–6), with a mean time of 24 h of postoperative mobilization. Only one surgical complication was recorded intraoperatively in the study group: the laceration of the peritoneum without bowel perforation, treated intraoperatively and which did not require a surgical revision. All patients were discharged home. Patients’ demographic and operative characteristics from both groups are summarized in Table 1 and Table 2. 

### 3.2. Clinical and Radiological Outcomes

All clinical outcomes in the study group improved significantly after surgery and remained stable during follow-up. Comparing the study and the control groups, the ODI scores improved from 51.2 ± 13.9 to 21.1 ± 6.2 at follow-up (*p* < 0.05). The SF-36 scores (preop 39.1 ± 5.9 vs. 71.3 ± 6.3 at follow-up, *p* < 0.05) and VAS scores (preop 8.1 ± 1.2 vs. 2.6 ± 0.9 at follow up, *p* < 0.05) improved significantly after surgery. However, there was a difference in the ODI score at the last follow-up in the study group compared to the control group (19.1 ± 6.1 vs. 22.5 ± 6.3, *p* = 0.045). The clinical outcomes of both groups are summarized in Table 3.

Concerning radiological results, LL (preop −36.9° ± 7.2 vs. −46.4° ± 8.1 at follow-up, *p* < 0.05 in the study group; preop −36.4° ± 6.8 vs. −45.9° ± 7.9 at follow-up, *p* < 0.05 in the control group), SL (preop −5.6° ± 4.3 vs. −9.8° ± 2.1 at follow-up, *p* < 0.05 in the study group; preop −5.9° ± 4.2 vs. −9.4° ± 2.0 at follow-up, *p* < 0.05 in the control group), and PI-LL mismatch (preop 16.9° ± 6.4 vs. 9.6° ± 2.8 at follow-up, *p* < 0.05 in the study group; preop 17.4° ± 6.9 vs. 9.8° ± 3.1 at follow-up, *p* < 0.05 in the control group) improved significantly after surgery and were maintained at follow-up in both groups without a significant difference between them. Similarly, FH (preop 12.9 ± 1.2 mm vs. 15.1 ± 1.1 at follow-up, *p* < 0.05 in the study group; preop 12.5 ± 1.3 mm vs. 14.6 ± 1.2 at follow-up, *p* < 0.05 in the control group) and DH (preop 5.3 ± 1.2 mm vs. 9.1 ± 1.0 mm at follow-up, *p* < 0.05 in the study group; preop 5.7 ± 1.3 mm vs. 9.0 ± 0.8 at follow-up, *p* < 0.05 in the control group) improved significantly after surgery in both groups without significant differences between groups.

**Figure 1:**
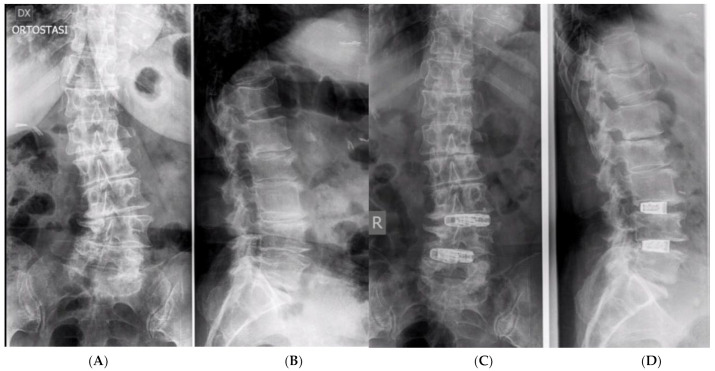
A 74-year-old woman. (**A**,**B**). Multiple DDD with left convex scoliosis and segmental L3-L5 kyphosis. (**C**,**D**) Postoperative X-rays showing two stand-alone expandable OLIF cages at L3-L4 and L4-L5.

**Figure 2:**
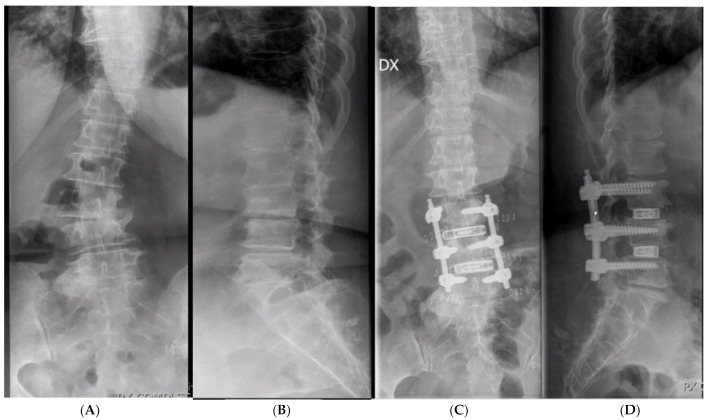
A 72-year-old women. (**A**,**B**) L3-L4 and L4-L5 DDD with right convex scoliosis and segmental L3-L5 kyphosis. (**C**,**D**) Postop X-rays showing L3-L4 and L4-L5 expandable OLIF cages supported by posterior instrumentation, with correction of the coronal plane. Note that expanded cages reach both lateral aspects of the epiphyseal ring with indirect foraminal decompression.

Contrarily, the segmental coronal Cobb angle (preop 12.9 ± 1.2 mm vs. 15.1 ± 1.1 at follow-up, *p* < 0.05 in the study group; preop 12.5 ± 1.3 mm vs. 14.6 ± 1.2 at follow-up, *p* < 0.05 in the control group) improved significantly after surgery in both groups with a significant difference between groups at follow-up (*p* = 0.02). The fusion rate at follow-up was higher in the study group (95%) compared to the control group (78.9%), *p* = 0.035. All patients’ radiological outcomes are summarized in Table 4, and two illustrative cases are presented in Figure 1 and Figure 2.

### 3.3. Complications and Reoperation Rate

Cage subsidence was demonstrated in 2 cages implanted in the study group (5%) and in 8 cages out of 38 in the control group (21.1%), with a significant difference between groups (*p* = 0.035). No postoperative major complications were recorded in both groups. One minor complication (3.6%) was observed in both groups: one superficial wound infection with complete resolution within 2 weeks. One patient in the study group (3.6%) and three patients in the control group (10.7%) with signs of cage subsidence and/or inconsistent arthrodesis, who continued to present persistent lower back pain, had a revision surgery with percutaneous pedicle screws in the segments involved in the first surgery.

## 4. Discussion

The ATP approach is a well-described surgical technique, directed toward the minimally invasive exposition of the antero-lateral aspect of the lumbar column, through the opening of retroperitoneal space with the patient in the right lateral decubitus position [14,15]. While for L5-S1, the ATP approach is comparable to ALIF in terms of the surgical corridor below the vascular carrefour, above L5, the ATP approach is directed to the aorto-psoas corridor. At L1-L2, the limit could be represented by an unfavorable working angle due to the rib cage, while inferiorly, the anatomy and course of the common left iliac vein could impede a safe approach to the L4-L5 or L5-S1 disc space [15,21,22]. Both are not absolute limits; in fact, with the skin incision around the 12th rib, it is possible to slide along the diaphragm and reach the L1-L2 space; conversely, with adequate experience, it is feasible to gently mobilize the left common iliac vein and create a sufficient surgical corridor to insert a cage at L5-S1. In any case, careful patient selection and preoperative planning, avoiding cases potentially complicated by the course of the left common iliac vein just on the disk, are critical factors to obtain good clinical results [16]. We excluded ATP approaches in the cases of patients with preoperative imaging demonstrating a common left iliac vein lying on the target disk [14,16]. We have already demonstrated that oblique cages inserted through an OLIF approach are particularly effective in correcting segmental kyphosis compared to straight cages inserted through a lateral trans-psoas approach in the case of DDD [1,23]. 

The results of the present study seem to confirm our previous findings. Both groups in the study showed similar results in terms of LL, SL, PI-LL mismatch, FH, and DH, and this is, in our opinion, due the possibility of incising the ALL with the OLIF approach. On the other hand, we have also demonstrated that in the case of severe segmental coronal imbalance, straight lateral cages inserted through a trans-psoas approach are able to achieve a major correction in the coronal plane, more than the one obtained with oblique cages inserted through an OLIF approach [1,4,22,23,24]. In this case, in fact, standard OLIF cages, positioned in the middle of the vertebral body, cannot cover the entire endplate and are not suitable for adequate coronal correction. Furthermore, the small footprint of the oblique cages, compared to those used for ALIF or LLIF, could be a reason for criticism, because it could reduce the possibility of obtaining solid arthrodesis. These are indeed the major criticisms to OLIF oblique cages, and they are the cause of frustration for surgeons dealing with such an approach [1,4,22,23]. Trying to overcome these limits, straight or slightly curved cages, similar to those used for the lateral approach, have been proposed for the ATP approach, but their use is limited by the challenging maneuvers needed to insert and rotate the cage through a such small window. Furthermore, to insert them with the correct orientation, a huge retraction of the psoas muscles is required, with postoperative pain and discomfort [17]. For such reasons, we developed a new ATP oblique expandable cage which has the advantages of both oblique insertion and small dimensions, typical of an OLIF standard cage, or, once opened, the final large footprint typical of a standard lateral cage. This new cage can be inserted easily through a standard small ATP approach, and it behaves as a large-footprint lateral cage. Moreover, reaching both lateral limits of the epiphyseal ring, it is able to significantly reduce the risk of subsidence, as our results seem to confirm [25,26,27]. To achieve clinical and surgical results, the choice of the right length, height, and lordosis of the cage is, however, always of utmost importance. A larger-footprint cage, able to reach both lateral limits of the epiphyseal ring, can biomechanically better support the anterior column and also facilitate interbody fusion. The standard technique of insertion of this new expandable cage is safe and easy. First, it forecasts the insertion of the closed cage up to contralateral side: at this stage, the a/p X-ray should show the distal part of the cage covering the contralateral pedicle. Then, the cage should be opened in a reverse fashion, reaching the ipsilateral pedicle, and the different sizes in length allows it to fit the right measure of the transversal axis of the endplate. The reverse opening is facilitated by the same route created by the cage insertion, and no particular forces must be applied to expand the cage.

### Limitations of the Study

There are some limitations to this study. First, the study compared prospective with historical data, and this was conducted by case selection and was not randomized. Second, the follow-up period was short and the sample of patients was relatively small. Lastly, the heterogeneity of procedures limits the interpretation of the data and its results. Nevertheless, future prospective randomized studies involving a long-term follow-up with a larger number of patients are required to clarify the advantages of this new expandable cage.

## 5. Conclusions

Our preliminary results seem to confirm that this expandable cage can obtain, at follow-up, more solid arthrodesis compared to the smaller oblique, standard OLIF cages. This characteristic could be due to the larger foot-print or the quality of 3D-printed titanium, which also guarantee a stronger grip between the cage surface and endplates with less risk of cage subsidence or mobilization. However, further studies with larger cohorts and longer follow-ups are needed to confirm these results.

## Figures and Tables

**Table 1:** Patient characteristics.

	Expandable Cage Group	Control Group	*p*-Value
**Total No. of Patients**	28	28	
Mean (SD) age, years (range)	64.2 ± 7.2 (42–81)	63.3 ± 7.2 (48–74)	0.64
Mean (SD) follow-up, months (range)	31.2 ± 10.8 (13–37)	34.2 ± (14–48)	0.08
**Sex**			
Female	12 (42.9%)	13 (46.4%)	0.79
Male	16 (57.1%)	15 (53.6%)	0.79
**ASA Classification**			
I	3 (10.7%)	4 (14.3%)	0.69
II	9 (32.2%)	10 (35.7%)	0.78
III	14 (50%)	11 (39.3%)	0.42
IV	2 (7.1%)	3 (10.7%)	0.64
V	0	0	1
Clinical presentation *			
Lower back pain	28 (100%)	28 (100%)	1
Radiculopathy	11 (39.3%)	12 (42.9%)	0.79
Neurogenic claudication	6 (21.4%)	8 (28.6%)	0.54
Comorbidity *			
Cardiovascular diseases	17 (60.7%)	19 (67.9%)	0.58
Diabetes mellitus	10 (35.7%)	7 (25%)	0.39
Obesity	8 (28.6%)	6 (21.4%)	0.54
Respiratory disease	6 (21.4%)	5 (17.9%)	0.74
Smokers	13 (46.4%)	15 (53.6%)	0.559

ASA, American Society of Anesthesiologists; * patients may have multiple clinical presentations and comorbidities.

**Table 2:** Operative characteristics.

	Expandable Cage Group	Control Group	*p* Value (Exp vs. Control)
**Level**			
L2-L3	6 (15%)	5 (13.2%)	
L3-L4	9 (22.5%)	7 (18.4%)	
L4-L5	21 (52.5%)	23 (60.5%)	
L5-S1	4 (10%)	3 (7.9%)	
Total levels treated	40	38	
**Levels treated**			
One level	19 (67.9%)	21 (75%)	
Two levels	6 (21.4%)	4 (14.3%)	
Three levels	3 (10.7%)	3 (10.7%)	
Radiological presentation *			
DDD	19 (67.8%)	18 (64.3%)	
Spondylolisthesis	12 (42.8%)	10 (43.7%)	
ASD	10 (35.7%)	9 (32.1%)	
**Type of surgery**			
Stand-alone	12 (42.9%)	9 (32.1%)	0.4
With posterior instrumentation	16 (57.1%)	19 (67.9%)	0.41
**Mean length of surgery (range)**	89 ± 14.8 min (60–210)	86 ± 15.1 min (50–190)	0.46
**Mean length of hospital stay (range)**	2 days (1–6)	2 days (1–4)	1
**Mean time of postoperative mobilization (range)**	1 day (1–4)	1 day (1–3)	1
**Intraoperative blood loss (range)**	100 ± 16.2 mL (40–200)	94 ± 14.8 mL (60–180)	0.15
**Complications**			
Superficial wound infection	1 (3.6%)	1 (3.6%)	1
Peritoneum perforation	1 (3.6%)	0	0.32
Subsidence °	2 (5%)	8 (21.1%)	**0.035**
**Reoperation Rate**	1 (3.6%)	3 (10.7%)	0.3

ASD, adjacent segment degeneration; DDD, degenerative disc disease; * patients may have multiple radiological presentations. In bold font, statistically significant results are shown; ° for a total of 40 levels in the first group and 38 levels in the second one.

**Table 3:** Clinical outcomes.

	Expandable Cage Group	Control Group	*p* Value (Exp vs. Control)
MEAN ± SD			
**Visual Analog Scale (VAS)**			
Preoperative	8.3 ± 1.1	7.9 ± 1.0	0.16
Postoperative (6 weeks)	3.3 ± 1.2	3.1 ± 1.3	0.55
Follow-up	2.7 ± 0.8	2.4 ± 0.9	0.19
*p* value (pre vs. follow-up)	**<0.05**	**<0.05**	
**Oswestry Disability Index (ODI)**			
Preoperative	53.2 ± 13.1	54.2 ± 12.8	0.77
Postoperative (6 weeks)	25.1 ± 8.1	26.5 ± 7.5	0.51
Follow-up	19.1 ± 6.1	22.5 ± 6.3	**0.045**
*p* value (pre vs. follow-up)	**<0.05**	**<0.05**	
**SF-36 (Physical and Mental)**			
Preoperative	40.1 ± 6.0	39.1 ± 5.7	0.53
Postoperative (6 weeks)	63.8 ± 7.7	63.6 ± 7.1	0.92
Follow-up	72.9 ± 6.4	70.4 ± 6.7	0.16
*p* value (pre vs. follow-up)	**<0.05**	**<0.05**	

SD, standard deviation; in bold font, statistically significant results are shown.

**Table 4:** Radiological outcomes.

	Expandable Cage Group	Control Group	*p* Value (Exp vs. Control)
	MEAN ± SD		
**Lumbar lordosis (LL) °**			
Preoperative	−36.9 ± 7.2	−36.4 ± 6.8	0.79
Postoperative (6 weeks)	−44.7 ± 8.0	−45.6 ± 8.2	0.68
Follow-up	−46.4 ± 8.1	−45.9 ± 7.9	0.82
*p* value (pre vs. follow-up)	**<0.05**	**<0.05**	
**Segmental lordosis (SL) °**			
Preoperative	−5.6 ± 4.3	−5.9 ± 4.2	0.79
Postoperative (6 weeks)	−10.1 ± 2.2	−10.6 ± 2.4	0.42
Follow-up	−9.8 ± 2.1	−9.4 ± 2.0	0.47
*p* value (pre vs. follow-up)	**<0.05**	**<0.05**	
**PI-LL mismatch °**			
Preoperative	16.9 ± 6.4	17.4 ± 6.9	0.78
Postoperative (6 weeks)	9.1 ± 3.0	9.3 ± 3.3	0.81
Follow-up	9.6 ± 2.8	9.8 ± 3.1	0.80
*p* value (pre vs. follow-up)	**<0.05**	**<0.05**	
**Coronal Cobb angle °**			
Preoperative	12.4 ± 1.1	11.9 ± 1.2	0.11
Postoperative (6 weeks)	16.2 ± 1.4	15.4 ± 1.3	**0.03**
Follow-up	15.4 ± 1.2	14.7 ± 1.0	**0.02**
*p* value (pre vs. follow-up)	**<0.05**	**<0.05**	
**Foraminal height (FH), mm**			
Preoperative	12.9 ± 1.2	12.5 ± 1.3	0.24
Postoperative (6 weeks)	16.0 ± 1.3	15.8 ± 1.5	0.60
Follow-up	15.1 ± 1.1	14.6 ± 1.2	0.11
*p* value (pre vs. follow-up)	**<0.05**	**<0.05**	
**Disc height (DH), mm**			
Preoperative	5.3 ± 1.2	5.7 ± 1.3	0.24
Postoperative (6 weeks)	9.6 ± 0.8	9.4 ± 0.9	0.38
Follow-up	9.1 ± 1.0	9.0 ± 0.8	0.68
*p* value (pre vs. follow-up)	**<0.05**	**<0.05**	
Fusion rate * (n, %)	38 (95%)	30 (78.9%)	**0.035**

* For a total of 40 levels in the first group and 38 levels in the second one. In bold font, statistically significant results are shown. ° degrees.

## Data Availability

The raw data supporting the conclusions of this article will be made available by the authors on request.
